# Abnormal Topological Organization of Structural Covariance Networks in Patients with Temporal Lobe Epilepsy Comorbid Sleep Disorder

**DOI:** 10.3390/brainsci13101493

**Published:** 2023-10-22

**Authors:** Shengyu Yang, Ying Wu, Lanfeng Sun, Yuling Lu, Kai Qian, Huimin Kuang, Jie Meng, Yuan Wu

**Affiliations:** Department of Neurology, The First Affiliated Hospital, Guangxi Medical University, Nanning 530021, China

**Keywords:** temporal lobe epilepsy, sleep disorder, structural covariance network (SCN), structural magnetic resonance imaging, graph theory

## Abstract

Objective: The structural covariance network (SCN) alterations in patients with temporal lobe epilepsy and comorbid sleep disorder (PWSD) remain poorly understood. This study aimed to investigate changes in SCNs using structural magnetic resonance imaging. Methods: Thirty-four PWSD patients, thirty-three patients with temporal lobe epilepsy without sleep disorder (PWoSD), and seventeen healthy controls underwent high-resolution structural MRI imaging. Subsequently, SCNs were constructed based on gray matter volume and analyzed via graph-theoretical approaches. Results: PWSD exhibited significantly increased clustering coefficients, shortest path lengths, transitivity, and local efficiency. In addition, various distributions and numbers of SCN hubs were identified in PWSD. Furthermore, PWSD networks were less robust to random and target attacks than those of healthy controls and PWoSD patients. Conclusion: This study identifies aberrant SCN changes in PWSD that may be related to the susceptibility of patients with epilepsy to sleep disorders.

## 1. Introduction

Epilepsy is one of the most common neurological disorders, and sleep disorder is a common comorbidity. Sleep disorders and epilepsy can coexist and impact each other in an individual. Seizures and interictal epileptic activity disrupt the normal sleep architecture by decreasing sleep efficiency and total sleep time and increasing sleep fragmentation, which in turn increases the risk of seizures, creating a vicious cycle [1,2]. Previous studies have revealed that people with epilepsy (28.9–51%) are more likely than the general population to develop sleep disorder, the risk being approximately 2–4 times greater than that for the general population [3,4]. Epileptic patients have a poorer quality of life than people without epilepsy, and sleep disorder increases the risk of daytime and night-time seizures and may lead to psychiatric comorbidities and cognitive impairment, leading to a further worsening in quality of life [5,6]. In addition, because seizures often occur during sleep, both epilepsy and sleep disorders may increase the risk of hypoxia and autonomic dysfunction during seizures, thereby increasing the risk of sudden death in patients with epilepsy [7].

Advanced functional and structural MRI methods can reveal more information about the disease than conventional MRI and are rapidly becoming strong tools for studying changes in brain structure and networks in neurological disorders. Studies show that thalamo-cortical networks are strongly associated with sleep/awake-related epileptogenesis in generalized and focal seizures [8]. Pathologically synchronized activity within the epileptogenic network is differentially regulated by specific alternating states of awake and sleep, and brain network properties also exhibit dynamic reorganization associated with awake and sleep [9,10]. However, there are still few structural or functional MRI studies of epilepsy and sleep, and we still know very little about the cerebral structural network changes in patients with temporal lobe epilepsy (TLE) and comorbid sleep disorder (PWSD). Chiosa et al. found that seizures during sleep and awake in patients with epilepsy present different structural networks and structural correlates [9]. The structural alterations that distinguish patients with seizures during sleep from those during waking involve cortical and subcortical brain areas, and abnormalities in these structures and networks may be associated with seizures in both sleep and waking states. However, their study focused on brain structure and networks alterations where seizures occur in different states and did not examine structural network changes in PWSD. Additionally, a complex brain network investigation utilizing graph theory might be more advantageous than a standalone local connectivity analysis for investigating the mechanisms of epilepsy and sleep disorder comorbidities because the pathophysiology of epilepsy is characterized by a wide range of brain regions and widespread network involvement.

The SCN uses correlation analysis of cross-sectional imaging data to reflect the correlation of structural covariates between brain regions in response to changes in specific environmental factors at very large time scales, allowing measurement of simultaneous morphological changes between brain regions undergoing common pathological processes [11,12]. Therefore, in the present study, we intended to assess network topology alterations in PWSD by structural covariance networks constructed via brain gray matter volume. We hypothesized that PWSD patients may have significant structural network alterations when compared with PWoSD patients or healthy controls.

## 2. Materials and Methods

### 2.1. Participants

The Pittsburgh sleep quality index (PSQI) is a 7-part patient self-rating questionnaire that is commonly performed to assess a subject’s sleep quality. The maximum total score of the questionnaire is 21, and a score > 5 indicates poor sleep quality, called sleep disorder [13,14]. Only patients who fulfilled the following criteria were included in the PWSD category, according to Nobili [2,6]: 1. patients diagnosed with TLE according to the 2017 ILAE criteria; 2. patients with a score > 5 for the PSQI. Individuals with other neurological disorders, severe systemic diseases, and brain lesions were barred from participating in this study. A gender- and age-matched cohort of TLE patients with a PSQI score ≤ 5 were enrolled as patients with TLE without sleep disorder (PWoSD). Additionally, 17 healthy participants were recruited in the control group. None of the participants suffered from serious systemic conditions or psychiatric disorders in the control group. The baseline demographics of all individuals are depicted in Table 1. A total of 34 PWSD and 33 PWoSD patients who attended the Department of Neurology’s outpatient clinic between December 2018 and December 2021 were recruited in this study. This study was approved by the Medical Ethics Committee of the First Affiliated Hospital of Guangxi Medical University (2023-E432-01).

### 2.2. MRI Data Acquisition

High-resolution 3D-T1 MRI data were obtained using a 12-channel head coil on a 3.0-T Siemens scanner (Siemens AG, Erlangen, Germany). To minimize participant head motion, foam padding was used. High-resolution 3D-T1 MRI data were collected using the following parameters: slice thickness = 1 mm; matrix size = 256 × 256; repetition time = 20 ms; echo time = 2.45 ms; flip angle = 9°.

### 2.3. Data Processing

#### 2.3.1. Data Pre-Processing

The Statistical Parametric Mapping 12 software (SPM 12, http://www.fil.ion.ucl.ac.uk/spm/) (accessed on 17 April 2021) and the computational anatomy plugins (CAT12, http://dbm.neuro.uni-jena.de/cat/) (accessed on 17 April 2021) toolbox running under MATLAB R2020a (Mathworks, Sherborn, MA, USA) were employed for 3D-T1 MRI data preprocessing. Diffeomorphic Anatomical Registration Through Exponential Lie Algebra (DARTEL) was used to normalize high-resolution 3D-T1 MRI data to the Montreal Neurological Institute (MNI) template space. They were subsequently segmented into white matter, cerebrospinal fluid, and gray matter via the CAT12 toolbox using a fully automated algorithm. Finally, according to the AAL90 atlas (Table S1), the average GMV of 90 brain regions was extracted with the “ROI Signal Extractor” implemented in DPABI [15] (Data Processing & Analysis for Brain Imaging toolbox; http://rfmri.org/dpabi) (accessed on 7 November 2021) for subsequent construction of the SCNs.

#### 2.3.2. SCN Construction

Using the nodes previously designed according to the AAL atlas, the SCNs were constructed for GMV by the graph analysis toolbox [16]. The 90 brain regions were defined as nodes to calculate the Pearson correlation coefficients between the GMV in individual regions after removing the confounding effects of gender, age, and total intracranial volume. For each group, a 90 × 90 adjacency matrix, R = [r_ij_] (i = 1 … *N*, j = 1 … *N*, *N* = 90), was established. To limit the noise impacts of spurious connections, we thresholded the adjacency matrix into a binary matrix within the network sparsity range of 0.19 to 0.49 with an interval of 0.02.

#### 2.3.3. Network Parameters

Several global and regional network characteristics were commonly used to characterize the topological organization of the GMV-based SCN. The small-world index, global efficiency, local efficiency, clustering coefficients, shortest path length, and transitivity were all global characteristics. The definitions of these network properties were the same as in previous studies [17,18]. In brief, small-worldness was calculated by dividing the normalized clustering coefficients by the normalized characteristic path lengths. The clustering coefficient was employed to characterize network segregation, that is, it quantified whether there was a potential connection between a node and its neighbors. The shortest path length was evidently employed to characterize the optimal path from a node to another node in the network. Global efficiency was used to describe the ability of a node and the rest of the nodes within the network to transmit information concurrently and measure whether a node has the ability to control the global network to spread information. Local efficiency can be described as the average efficiency of each node’s domain subgraph in the network, and it was frequently used as a measure of network error tolerance.

Local parameters, on the other hand, comprised nodal clustering coefficients, nodal efficiency, betweenness, and degrees. Betweenness was defined as the ratio of all shortest paths going through a network node to all shortest paths. The degrees were used to characterize the number of connections a node has to the rest of the network’s nodes.

#### 2.3.4. Network Hubs

Network hubs are nodes that are instrumental in regulating the flow of information across a network. Herein, a node was designated a hub if its degree was more than one and a half standard deviations above the mean value [18]. The hubs of the two groups were visualized via the BrainNet viewer [19].

#### 2.3.5. Degree Distribution

The degree distribution is the probability distribution or frequency distribution of node degrees in the network. A previous study has established that the SCN of humans fulfills an exponentially truncated power-law distribution [20]. The expression of this degree distribution is as follows: $P(d)=d^{1/k}*{exp}^{\left(-\frac{d}{dc}\right)}$, where P(d) represents the odds of each region degree (d) in the network, k represents the power exponent, and dc represents the cut-off degree. The probability of high-degree nodes being greater than the dc cut-off decays exponentially [21]. To analyze differences in the degree distribution in the three groups, the log-rank test was performed.

#### 2.3.6. Network Robustness

A network’s resilience can be assessed by measuring the proportional decrease in the largest connected component in random or targeted attacks. Herein, random network failure was simulated by the random removal of one node from the network. Subsequently, network stability was assessed by estimating the size of the remaining largest connected component [21]. The average metrics of the remaining network were obtained through a 1000-times simulation in this study. As for the targeted attack, the same process was applied, that is, the nodes were removed from the network in rank ordering of descending nodal degrees [21]. Afterward, the between-difference of network robustness was measured during each attack by calculating the area under the curve (AUC).

#### 2.3.7. Statistical Analysis

An independent two-sample *t*-test using the Statistical Package for the Social Sciences (SPSS), version 22.0, was performed to analyze differences in age, seizure duration, number of antiepileptic medicines, total intracranial volume, and total GMV. The chi-squared test was used to estimate the difference in gender and seizure frequency. *p* < 0.05 was used as the significance criterion for group differences.

To estimate differences in all network characteristics between the groups, a non-parametric permutation test with 1000 repetitions was used [18,22]. For each replicate, each subject’s corrected GMV value was randomly assigned to two new groups with the following number as the original group. Subsequently, the between-group differences of network parameters at a range of network sparsities (0.19–0.49, step 0.02) were recalculated, resulting in a permutation distribution of differences under the null hypothesis. The actual difference between groups was placed in the corresponding permutation distribution, and a *p*-value was computed from the position of the percentile. To evaluate between-group variations in all sparsities, an AUC summary measure was performed [18]. *p* < 0.05 was established as the significance criterion for between-group differences in network characteristics.

## 3. Results

### 3.1. Clinical Characteristics

No significant differences were observed regarding gender and age between the three groups (*p* > 0.05) (Table 1). Seizure frequency, seizure duration, and amount of antiseizure medications did not differ significantly between PWSD and PWoSD (*p* > 0.05) (Table 1). Both groups were treated with antiepileptic drugs, including one or more of lamotrigine, oxcarbazepine, carbamazepine, levetiracetam, valproic acid, and topiramate. The main sleep disorders in PWSD were daytime dysfunction (82.35%), decreased sleep quality (76.47%), difficulty falling asleep (67.65%), sleep disturbances (55.88%), reduced sleep time (32.35%), decreased sleep efficiency (26.47%), and use of hypnotic drugs (8.8%).

### 3.2. Global Network Analysis

**Figure 1:**
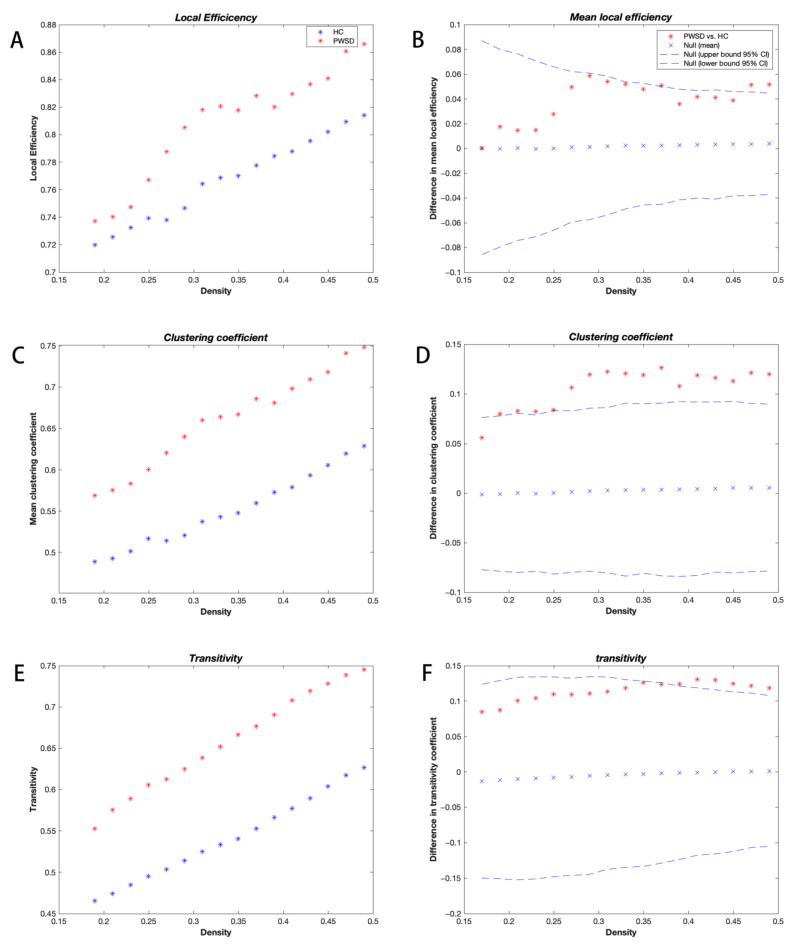
Changes in local efficiency (**A**,**B**), clustering coefficients (**C**,**D**), and transitivity (**E**,**F**) and between-group differences were measured in relation to network density for participants with PWSD and healthy controls. Between-group differences are denoted by * symbols, and * symbols located outside the 95% confidence intervals (dashed lines) indicate network densities with significant differences at *p* = 0.05. Positive numbers signify higher PWSD values compared to healthy controls, whereas negative values indicate the opposite.

**Figure 2:**
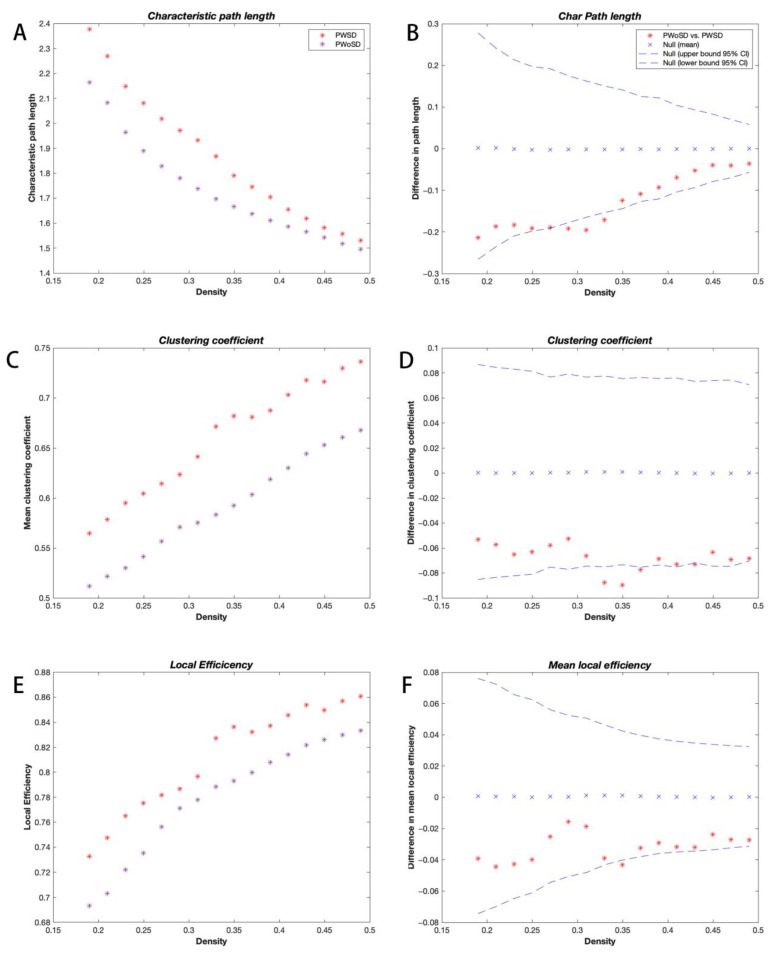
Changes in shortest path lengths (**A**,**B**), clustering coefficients (**C**,**D**), and local efficiency (**E**,**F**) and between-group differences were measured in relation to network density for participants with PWSD and PWoSD. Between-group differences are denoted by * symbols, and * symbols located outside the 95% confidence intervals (dashed lines) indicate network densities with significant differences at *p* = 0.05. Positive numbers signify higher PWSD values compared to healthy controls, whereas negative values indicate the opposite.

The binary adjacency matrices of the two groups revealed powerful correlations among most brain regions. The three groups’ SCNs displayed a small-world organization at all network sparsities with small-world indices greater than 1, but the small-world indices of the three groups showed no significant differences. However, PWSD displayed significantly increased local efficiency (*p* = 0.045; Figure 1A,B), clustering coefficients (*p* = 0.009; Figure 1C,D), and transitivity (*p* = 0.031; Figure 1E,F) compared with the healthy controls. In addition, when compared with PWoSD, PWSD showed significantly elevated shortest path lengths (*p* = 0.028; Figure 2A,B), clustering coefficients (*p* = 0.025; Figure 2C,D), and local efficiency (*p* = 0.047; Figure 2E,F).

### 3.3. Regional Network Analysis

The local network characteristics of the three groups were compared in addition to the global network metrics. After multiple-comparison correction, the disparities in regional network characteristics between the three groups were no longer present.

### 3.4. Network Hub Analysis

**Figure 3:**
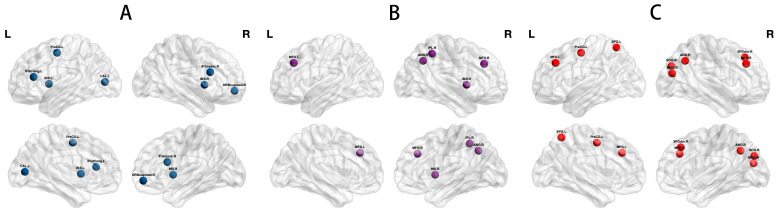
Network hubs in the three groups. Seven degree-based hubs were observed in healthy controls (**A**), while PWoSD showed five degree-based hubs (**B**), and PWSD exhibited eight degree-based hubs (**C**). Abbreviations are listed in Table S1.

Degree-based network hubs were confirmed in the three groups in varying numbers and distributions. Based on the degree, eight hubs were identified in PWSD, five hubs in PWoSD, and seven hubs in healthy controls (Figure 3) (Table 2).

### 3.5. Degree Distribution Analysis

**Figure 4:**
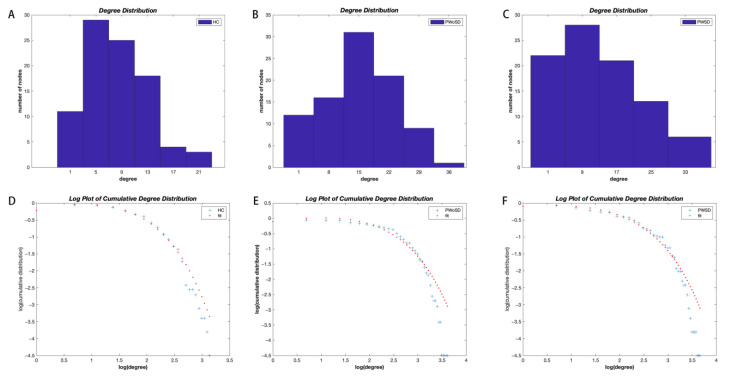
Degree distribution histograms for healthy controls (**A**), PWoSD (**B**), and PWSD (**C**). The log–log plots of the cumulative degree distributions of the networks of healthy controls (**D**), PWoSD (**E**), and PWSD (**F**).

The degree distributions of the three groups conformed to an exponentially truncated power-law distribution (Figure 4). The power exponent (k) was 1.1687, 1.3032, and 1.5532, while the dc was 10.4879, 9.3077, and 4.5241 for PWSD, PWoSD, and healthy controls, respectively. The fit of PWSD was 0.9834, for PWoH it was 0.9721, and for healthy controls it was 0.9784. The degree distributions of the three groups of brain networks were significantly different. PWSD had more high-degree regions compared to healthy controls (*p* = 0.0004), but there was no significant difference compared to PWoSD (*p* = 0.7).

### 3.6. Network Robustness Analysis

**Figure 5:**
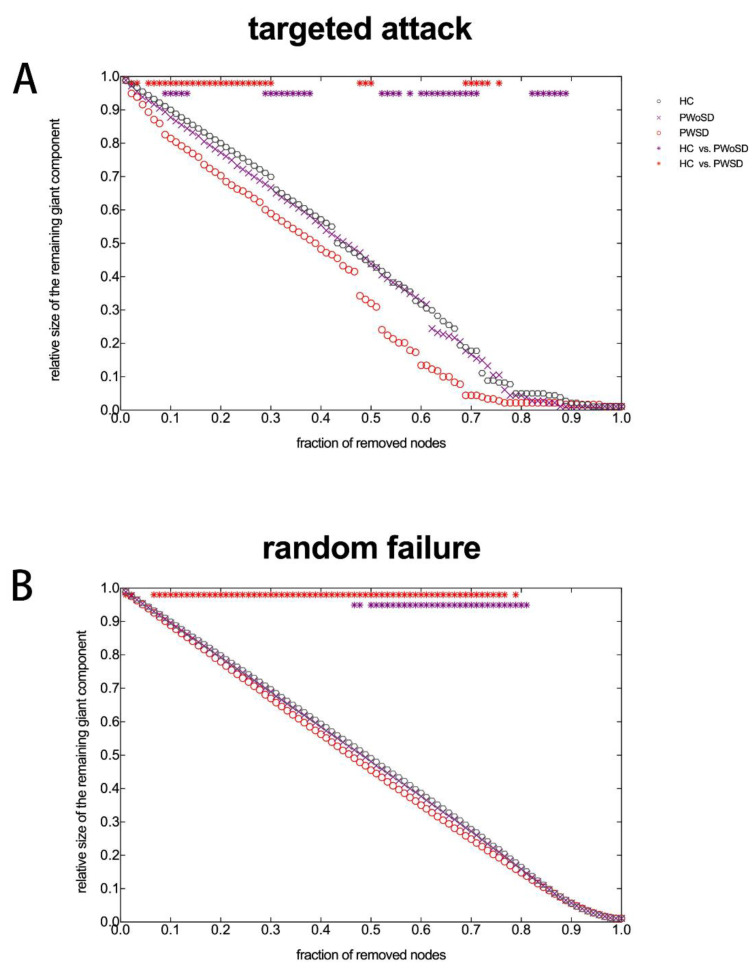
The robustness of a network to targeted attacks (**A**) and random failure (**B**). The network’s resilience increases as the remaining giant component’s relative size increases. Red stars represent significant between-group differences in network robustness in PWSD and healthy controls. Purple stars represent significant between-group differences in network robustness in PWSD and PWoSD.

Network robustness analysis revealed significant differences in the ability of the three groups to recover from targeted attacks and random attacks in the proportion of all deleted nodes (Figure 5). AUC analysis confirmed that PWSD was significantly less robust to both targeted attacks and random attacks compared to healthy controls and PWoSD.

## 4. Discussion

This study used gray matter volume to construct structural covariance networks to explore the effects of sleep disorders on brain networks in patients with TLE. Our investigation revealed that PWSD patients had significantly altered structural network topological characteristics, numbers and distributions of central hubs, and network robustness, suggesting a disrupted topological organization in epilepsy comorbid sleep disorder.

The functional and anatomical connection networks of the human brain were shown to be more “small-world” than random networks [23,24,25], as evidenced by their similar normalized path lengths and greater normalized clustering coefficients. Small-world networks enable far more effective information transfer in regional or global brain areas [26]. The results of this study show that all three groups exhibit small-world features, indicating that PWoSD and PWSD both maintain the small-world attribute. However, the clustering coefficient and transitivity were significantly increased in PWSD compared with healthy controls. In addition, compared with PWoSD, PWSD displayed significantly higher clustering coefficients and shortest path lengths. This outcome indicated significantly worsened local segregation and weaker network integration in PWSD, signaling that suboptimal reorganization of the structural network occurred and towards a regularized network configuration [21,27]. Decreased global integration in PWSD may reflect deficits or delays in teleconnectivity.

This study also revealed increased local efficiency in PWSD compared to the other two groups. Short-range connections between neighboring brain areas that control network fault tolerance or modular information processing are primarily linked to local efficiency. According to the study, the better the network’s local efficiency, the more fault-tolerant it is against external attacks [28]. The improvement in local efficiency may be connected to the modification of the brain’s structural function [29]. Additionally, it has been proposed that the improved local efficiency of brain networks may result from a network’s compensatory adaptation brought on by sleep limitation or deprivation [30,31]. Consequently, we postulate that the higher local efficiency in PWSD may be a compensation mechanism set off by the remodeling of brain structures brought on by the interplay of recurrent seizures and sleep disturbances, making their networks more forgiving and lessening the detrimental impacts of the condition.

Although both PWSD and PWoSD exhibit small-world networks, significant increases in clustering coefficients, shortest path lengths, and local efficiency disrupt the balance of local functional isolation and global integration in PWSD, leading them tend to develop from small-world networks to regularized networks. It has been shown that after the onset of seizures, the path lengths and clustering coefficients of brain networks in epileptic patients tend to increase and that the path lengths are particularly pronounced, moving toward regularized networks until the termination of seizures [32,33,34]. Previous graph-theoretic studies also reported significant increases in shortest path lengths and local efficiency in patients with sleep disorders [35,36]. Additionally, it has been indicated that sleep is crucial for the maintenance of small-world networks [37,38]. Thus, the structural brain network we observed in PWSD tends to develop from a small-world network to a regular network, possibly as a consequence of the combined effect of recurrent seizures and sleep disorders.

This study identified network hubs with different distributions and numbers based on nodal degrees among the three groups, suggesting a reorganization of network hubs in PWSD. The specific network hubs in PWSD were located in the dorsolateral superior frontal gyrus, superior occipital gyrus, middle occipital gyrus, and superior parietal gyrus compared to healthy controls and PWoSD. Previous studies have reported altered functional activation in the prefrontal cortex, frontoparietal, insula, and occipital lobes in sleep deprivation [30,39,40,41]. Functional connectivity between the lateral orbitofrontal cortex and regions of the thalamus, middle frontal gyrus, precentral gyrus, insula, superior temporal gyrus, and middle occipital gyrus correlates with PSQI scores [42]. Furthermore, it has been reported that a unique neuronal circuit exists between the prefrontal cortex and the reticular nucleus of the thalamus that promotes wakefulness [43] and that an overactive prefrontal cortex may lead to difficulties in sleep initiation [44]. We therefore hypothesized that network hub reorganization in PWSD patients may be closely related to their sleep disorders. However, whether this network hub reorganization is the cause or the outcome of sleep disorders, or both, is not yet known.

It has been revealed that the brain network follows an exponentially truncated power-law distribution [20,45], and this distribution pattern suggests that the brain network consists of fewer connected nodes and more connected hub nodes. This network configuration may withstand targeted attacks better than scale-free networks [46]. However, this study found that PWSD’s network was less resilient to both random and targeted attacks than the networks of the other two groups. The topological characteristics of a network have a significant impact on how resilient it is against random or targeted assaults. Nodes with high node degrees are tightly interconnected, and when one of them is removed the other nodes continue to keep the maximum connectivity of the network and thus are relatively stable against targeting attacks. However, since nodes with low node degrees tend to interconnect with nodes with low node degrees, the network is vulnerable to network fragmentation under random assaults. The distribution cut-off observed in this study was slightly higher in PWSD than in the other two groups, i.e., there were more nodes with high degree connections in the SCN of PWSD. In addition, the network of PWSD tended to be configured from a small-world network to a regularized network, which is less resilient to random attacks, suggesting that the SCN of PWSD may be less tolerant to a random attack [47].

The effects of antiseizure medications may be improved by customizing seizure preventive treatments to specific sleep-related seizure patterns, resulting in stronger control during seizure-prone phases. Therefore, a more extensive study on the monitoring of changes in network topological features in PWSD, in conjunction with long-term data, including EEG, functional, and structural MRI data, may be required in the future.

In addition to the SCN construction method used in this study, cube-based and distance-based methods are two other methods for constructing SCNs. The cube-based method constructs an SCN by partitioning the subject’s gray matter into cubes of 6 mm^3^ in size [48], defining each cube as a node, and calculating the similarity of each cube as an edge, which is a relatively good method for the construction of individual SCNs. However, it is analyzed in native space, not in standard space, and the number of nodes cannot be kept constant. Instead, we would like to focus on the network topology with the same number of nodes under the anatomical atlas for comparison with other studies. The distance-based approach involves adding patient-constructed individual brain networks to healthy subjects [49]. This is a novel approach, but a potential limitation of this method is that it requires a minimum number of participants (*n* = 25–30) to extract stable individual differences. In future studies, we will consider combining these three methods to study network alterations in patients.

There are several limitations to this study that should be mentioned. For starters, the sample size of the current study was small. As a result, the structural network changes revealed in PWSD need to be verified in a larger sample. Secondly, the structural network was constructed at the group level and therefore could not be correlated with PSQI scores for analysis. Finally, this study was a cross-sectional investigation in which we were unable to observe dynamic changes in the structural network of the brain in PWSD.

In conclusion, this study used SCNs to reveal the alterations in nodal degrees and nodal hubs in multiple brain regions and the abnormal reorganization of structural brain networks in PWSD, as well as decreased network robustness, allowing us to better understand the neuroanatomical and pathophysiological mechanisms at the network level that predispose epileptic patients to comorbid sleep disorders.

## Figures and Tables

**Table 1:** Demographic data of the study groups.

	Healthy Controls	PWoSD	PWSD	*p*-Value
Number of participants	17	33	34	-
Age (year/mean ± SD)	25.18 ± 3.01	22.30 ± 7.30	26.26 ± 9.27	0.10 ^a^
Gender (male/female)	9/8	21/12	15/19	0.28 ^b^
Handedness	22R	32R	25R	-
Seizure duration (year/mean ± SD)	-	7.15 ± 5.44	7.38 ± 6.19	0.87 ^c^
Seizure frequency (N/%)				
Seizure-free > 1 year	-	5(15.15)	3(8.82)	0.42 ^b^
1–2 per year	-	7(21.21)	2(5.88)	0.07 ^b^
1–5 in last 6 months	-	3(9.09)	2(5.88)	0.62 ^b^
1–5 per month	-	13(39.39)	17(50.00)	0.38 ^b^
>5 per month	-	5(15.15)	10(29.41)	0.16 ^b^
Period of seizure (N/%)				
Sleep	-	7(21.21)	15(44.12)	0.046 ^b^
Awake	-	17(51.51)	6(17.65)	0.004 ^b^
Both sleep and awake	-	9(27.27)	13(38.24)	0.34 ^b^
PSQI (mean ± SD)	3.47 ± 1.18	3.24 ± 1.66	9.06 ± 2.16	<0.0001 ^a^
ASMs (number/mean ± SD)	-	1.49 ± 0.80	1.59 ± 0.70	0.57 ^c^

PWoSD: patients with temporal lobe epilepsy without sleep disorder; PWSD: patients with temporal lobe epilepsy with sleep disorder; SD: standard deviation; R: right; ASMs: antiseizure medications; ^a^: one-way ANOVA; ^b^: chi-squared test; ^c^: independent two-sample *t*-test.

**Table 2:** Degree-based hub distributions in each group.

Hub Regions	Abbreviation
Healthy controls	
L precental gyrus	PreCG.L
R inferior frontal gyrus, opercular part	IFGoperc.R
L inferior frontal gyrus, triangular part	IFGtriang.L
L insula	ORBsupmed.R
R insula	INS.L
L calcarine fissure and surrounding cortex	INS.R
PWoSD	
L middle frontal gyrus	MFG.L
R middle frontal gyrus	MFG.R
R insula	INS.R
R inferior parietal, but supramarginal and angular gyri	IPL.R
R angular gyrus	ANG.R
PWSD	
L precental gyrus	PreCG.L
R superior frontal gyrus, dorsolateral	SFGdor.R
L middle frontal gyrus	MFG.L
R middle frontal gyrus	MFG.R
R superior occipital gyrus	SOG.R
R middle occipital gyrus	MOG.R
L superior parietal gyrus	SPG.L
R angular gyrus	ANG.R

PWoSD: patients with temporal lobe epilepsy without sleep disorder; PWSD: patients with temporal lobe epilepsy and comorbid sleep disorder; L: left; R: right.

## Data Availability

Data is unavailable due to privacy or ethical restrictions.

## Supplementary Materials

The following supporting information can be downloaded at: https://www.mdpi.com/article/10.3390/brainsci13101493/s1, Table S1: Names and indices of the AAL 90 parcellated brain regions.
