# Supplementary material for: Abnormal Topological Organization of Structural Covariance Networks in Patients with Temporal Lobe Epilepsy Comorbid Sleep Disorder
Source: Brain Sci. 2023 Oct 22;13(10):1493. doi: 10.3390/brainsci13101493 (PMC10605209; doi:10.3390/brainsci13101493)
Supplement: Supplementary file 1 [file brainsci-13-01493-s001.zip › brainsci-2624754-supplementary.pdf]

Table S1 Names and indices of the AAL 90 parcellated brain regions

Region	Abbreviations	Index (left)	Index (right)
Precentral gyrus	PreCG	1	2
Superior frontal gyrus	SFGdor	3	4
Orbitofrontal cortex (superior)	ORBsupb	5	6
Middle frontal gyrus	MFG	7	8
Orbitofrontal cortex (middle)	ORBmid	9	10
Inferior frontal gyrus (opercular)	IFGoperc	11	12
Inferior frontal gyrus (triangular)	IFGtriang	13	14
Orbitofrontal cortex (inferior)	ORBinf	15	16
Rolandic operculum	ROL	17	18
Supplementary motor area	SMA	19	20
Olfactory	OLF	21	22
Superior frontal gyrus (medial)	SFGmed	23	24
Orbitofrontal cortex (medial)	ORBmed	25	26
Rectus gyrus	REC	27	28
Insula	INS	29	30
Anterior cingulate gyrus	ACG	31	32
Middle cingulate gyrus	MCG	33	34
Posterior cingulate gyrus	PCG	35	36
Hippocampus	HIP	37	38
Parahippocampal gyrus	PHG	39	40
Amygdala	AMYG	41	42
Calcarine cortex	CAL	43	44
Cuneus	CUN	45	46
Lingual gyrus	LING	47	48
Superior occipital gyrus	SOG	49	50
Middle occipital gyrus	MOG	51	52
Inferior occipital gyrus	IOG	53	54
Fusiform gyrus	FFG	55	56
Postcentral gyrus	PoCG	57	58
Superior parietal gyrus	SPG	59	60
Inferior parietal lobule	IPL	61	62
Supramarginal gyrus	SMG	63	64
Angular gyrus	ANG	65	66
Precuneus	PCUN	67	68
Paracentral lobule	PCL	69	70
Caudate	CAU	71	72
Putamen	PUT	73	74
Pallidum	PAL	75	76
Thalamus	THA	77	78
Heschl gyrus	HES	79	80

Superior temporal gyrus	STG	81	82
Temporal pole (superior)	TPOsup	83	84
Middle temporal gyrus	MTG	85	86
Temporal pole (middle)	TPOmid	87	88
Inferior temporal gyrus	ITG	89	90
